# Soccer-based promotion of voluntary medical male circumcision: A mixed-methods feasibility study with secondary students in Uganda

**DOI:** 10.1371/journal.pone.0185929

**Published:** 2017-10-09

**Authors:** George Miiro, Jeff DeCelles, Rwamahe Rutakumwa, Jessica Nakiyingi-Miiro, Philip Muzira, Wilber Ssembajjwe, Saidat Musoke, Lorna J. Gibson, Rebecca B. Hershow, Suzanna Francis, Belen Torondel, David A. Ross, Helen A. Weiss, On behalf of the MENISCUS project

**Affiliations:** 1 Uganda Virus Research Institute, Entebbe, Uganda; 2 Grassroot Soccer, Cape Town, South Africa; 3 University of North Carolina Gillings School of Global Public Health, Chapel Hill, North Carolina, United States of America; 4 Medical Research Council/Uganda Virus Research Institute Uganda Research Unit on AIDS, Entebbe, Uganda; 5 Medical Research Council Tropical Epidemiology Group, Department of Infectious Disease Epidemiology, London School of Hygiene and Tropical Medicine, London, United Kingdom; 6 Department of Disease Control, London School of Hygiene and Tropical Medicine, London, United Kingdom; 7 Department of Maternal, Newborn, Child and Adolescent Health, World Health Organisation, Geneva, Swizterland; Cardiff University, UNITED KINGDOM

## Abstract

The Ugandan government is committed to scaling-up proven HIV prevention strategies including safe male circumcision, and innovative strategies are needed to increase circumcision uptake. The aim of this study was to assess the acceptability and feasibility of implementing a soccer-based intervention (“Make The Cut”) among schoolboys in a peri-urban district of Uganda. The intervention was led by trained, recently circumcised “coaches” who facilitated a 60-minute session delivered in schools, including an interactive penalty shoot-out game using metaphors for HIV prevention, sharing of the coaches’ circumcision story, group discussion and ongoing engagement from the coach to facilitate linkage to male circumcision. The study took place in four secondary schools in Entebbe sub-district, Uganda. Acceptability of safe male circumcision was assessed through a cross-sectional quantitative survey. The feasibility of implementing the intervention was assessed by piloting the intervention in one school, modifying it, and implementing the modified version in a second school. Perceptions of the intervention were assessed with in-depth interviews with participants. Of the 210 boys in the cross-sectional survey, 59% reported being circumcised. Findings showed high levels of knowledge and generally favourable perceptions of circumcision. The initial implementation of Make The Cut resulted in 6/58 uncircumcised boys (10.3%) becoming circumcised. Changes made included increasing engagement with parents and improved liaison with schools regarding the timing of the intervention. Following this, uptake improved to 18/69 (26.1%) in the second school. In-depth interviews highlighted the important role of family and peer support and the coach in facilitating the decision to circumcise. This study showed that the modified Make The Cut intervention may be effective to increase uptake of safe male circumcision in this population. However, the intervention is time-intensive, and further work is needed to assess the cost-effectiveness of the intervention conducted at scale.

## Introduction

The annual number of people newly infected with HIV has remained stable in the last few years, with an estimated 1.9 million new infections globally in 2015 [1]. In Uganda, approximately 7% of the adult population is estimated to be living with HIV, with an estimated 0.8% incidence in the general population in 2013 [2]. The Ugandan government is committed to scaling-up proven HIV prevention strategies including provision of voluntary medical male circumcision (VMMC), which reduces a male’s risk of acquiring HIV infection by about 60% [3]. Uganda is one of 14 VMMC priority countries in Sub-Saharan Africa, due to high HIV incidence and low circumcision prevalence, and an estimated 2.7 million VMMCs were performed between 2008 and 2015 [4]. However, further demand-creation efforts are needed to reach the target of 4.2 million men circumcised by 2025 (80% coverage) [5–7].

The greatest impact of VMMC on HIV incidence over a 15-year period will result from focusing on uptake among boys and men aged 10–19 years [8]. To increase VMMC uptake among young men, demand-creation interventions have included activities in schools and sport-based activities [9, 10]. For example, the Ugandan focus on HIV prevention among the youth includes a sport-based national campaign “Protect the Goal” launched by the President in 2014 to focus on the power of sports to unite Ugandans towards the goal of an AIDS-free generation [11]. In 2012, Grassroot Soccer (GRS), a sport-based HIV prevention organization initiated in Zimbabwe, developed a single-session VMMC demand-creation intervention called Make The Cut in which trained, recently circumcised “coaches” facilitate a 60-minute educational session with male adolescent students. The intervention uses a penalty shoot-out game as a metaphor for HIV prevention, including the role of condoms and VMMC, and coaches share their own personal stories of VMMC to motivate boys to undergo the procedure. Two cluster-randomized trials of the Make The Cut intervention in Bulawayo, Zimbabwe produced promising evidence of its effect, especially among school-going youths, which found strong evidence that the intervention increased VMMC uptake from 4.6% to 12.2% (odds ratio (OR) = 2.65; 95% confidence interval (95% CI):1.19–5.86) [9, 12].

Whilst the Make The Cut intervention produced promising results of VMMC demand-creation among school-going youth in Zimbabwe, additional studies are needed to evaluate the effectiveness in other settings. In this paper, we report findings of a mixed-methods feasibility study undertaken in four secondary schools in Uganda as part of the Menstrual Hygiene and Safe Male Circumcision Promotion in Ugandan Schools (MENISCUS) study. The aim of this study was to evaluate the perception, knowledge and attitudes towards VMMC among schoolboys in Entebbe sub-District, Uganda, and to assess the feasibility and acceptability of the Make The Cut intervention and its potential effect on VMMC uptake. Findings from this study will inform future scale-up and research of VMMC promotion in Uganda.

## Methods

We used a mixed-methods (quantitative and qualitative) approach to explore the feasibility and acceptability of Make The Cut in Uganda.

### Study setting

The study was conducted in Entebbe sub-district, a peri-urban area within Wakiso district, Uganda. The sub-District contains 13 registered secondary schools, including three government-sponsored schools. Four secondary schools were purposively selected for the study. The schools included one government school providing Universal Secondary Education (USE—a scheme for providing free education to secondary school students to improve education access to children from low-income families); one government non-USE school; and two private schools (one catering to predominantly high-income families and one to low-income families). Prior to initiating the research, a stakeholders meeting was held in March 2015 with the Entebbe Municipality authorities in the education and health sectors, to discuss the school selection, study purpose, objectives, procedures and duration.

### Quantitative cross-sectional survey

In October and November 2015, a cross-sectional survey was conducted in the four selected schools. All male students in Forms 2 and 3 (median ages 16–17 years) who were present on the day of the visit were eligible. All boys present were asked for written informed consent for the quantitative and qualitative studies if aged ≥18 years. If aged <18 years, boys were asked for written informed assent after their parents provided consent. For those who assented/consented, a self-completed paper questionnaire was administered in the classroom, with a facilitator present to provide advice to students on how to complete the questionnaire, if required. The questionnaire included demographic and socio-economic characteristics; parental characteristics; school attendance; knowledge, attitudes and perceptions of safe male circumcision and HIV/AIDS, and sexual behaviour.

### Qualitative study

Qualitative data were collected using in-depth interviews (IDIs) among boys participating in the Make The Cut intervention. Five boys who were circumcised following the intervention were purposively selected for IDIs as were five boys who registered an interest in VMMC during the intervention, but remained uncircumcised by the end of the study. The aim of these interviews was to explore the perception and acceptability of Make The Cut, perceptions of VMMC, suggestions for improvements to the intervention, and teaching about VMMC and reproductive health in the school curriculum.

### Make The Cut intervention

The substudy on the feasibility and acceptability of the Make The Cut intervention for increasing VMMC uptake was conducted in the two low SES schools between April and July 2016. The intervention has been described previously [9]. Briefly, it consists of a 60-minute session delivered in schools by a trained facilitator or ‘coach’–a young man trained by Grassroot Soccer as an HIV educator who is self-reported as being circumcised. The session includes an interactive penalty shoot-out game, a personal story shared by the coach, and a group discussion. Key messages communicated focus on the health benefits of VMMC, including improved hygiene and protection from sexually transmitted infections. In sharing the story of their own decision to become circumcised and discussing their experience, coaches build personal connections with participants and address barriers to seeking VMMC, such as fear of pain during and after the surgery. In the week following intervention delivery, coaches follow-up with boys who, after going through the Make The Cut intervention, said that they wanted to be circumcised. Coaches communicate with boys either in-person (at home or school), or by phone or social media platforms such as SMS or WhatsApp. Coaches schedule a time to escort these boys to the VMMC clinic and stayed with them through the duration of the procedure. Following circumcision, the coaches continued to be in contact for two weeks, with additional home visits if requested following this period—for example, to deliver additional painkillers as needed.

For this feasibility study, we iteratively adapted the intervention over three stages:

1. Revisions based on review of the Make The Cut process evaluation from Zimbabwe [13] and secondary research of VMMC demand-creation interventions.
2. Revisions based on a workshop held in Uganda with key stakeholders in March 2016.
3. Revisions based on VMMC uptake data and observations from the first implementation school.

A process evaluation of Make The Cut in Zimbabwe identified that establishing a strong relationship between the coach and interested participants was crucial for maintaining boys’ motivation to undergo circumcision. Key factors for establishing this relationship were the recruitment and training of circumcised males as coaches, conducting phone-based follow-up calls with participants, and facilitating coach accompaniment of boys to the clinic. These components were included in the Ugandan intervention, while other components found to be less effective were excluded (e.g. mass SMSs and posters featuring professional soccer players).

### Data analysis

Statistical analyses were conducted using Stata 14 (StataCorp, TX, USA). Socio-demographic characteristics, knowledge and perceptions about circumcision, by circumcision status, were described by school. A binary “urban socioeconomic status” variable was developed using the answers to the questions on electricity, running water inside the house, and ownership of a refrigerator, TV, and functioning car. Students answered five true/false questions on basic HIV information to estimate their level of HIV knowledge. Questions on the perceptions of circumcision were scored on a 5-point Likert scale, and analysed as the proportion who either agreed or strongly agreed with the statement given. Logistic regression was used to calculate adjusted prevalence ratios (aPRs) and 95% confidence intervals (CIs) for the association with being circumcised and other demographic and lifestyle factors [14]. Factors that were found to have a *p*<0.15 in the univariable analysis were included in an initial multivariable logistic regression model. Factors that had a p<0.1 were retained in the final analysis. An *a priori* decision was taken to include type of school and religion in all models as these were known to be associated with circumcision status.

For the qualitative analysis of in-depth interviews with boys who participated in the Make The Cut intervention, a thematic content analysis was led by the senior social scientist with two male research assistants. Each research assistant initially reviewed an interview transcript. In addition, a transcript was assigned to both research assistants to test inter-rater reliability. This was followed by iterative discussions of emerging themes and sub-themes and development of a coding framework. The framework was further refined as more transcripts were analysed, by classifying key themes and sub-themes in a matrix, and capturing participant narratives accordingly to illustrate each theme. During and after the data collection phase, team members held weekly or bi-weekly meetings to review progress with data analysis, to discuss and resolve issues and challenges arising from the analysis, and to ensure consistency in the use of the coding framework.

### Ethics

The study was approved by the Ethics Committees of the Uganda Virus Research Institute (GC/127 /15/04/508), the Uganda National Council for Science and Technology (HS/1810), and the London School of Hygiene and Tropical Medicine (Ref 9682).

## Results

### Demographic data

Of the 210 boys present in Forms 2 or 3 on the day of the survey, all provided written informed assent with parental consent, and completed the questionnaire. Socioeconomic characteristics varied, with one government and one private school being of low SES and the other two of high SES. The majority of boys were day students (84%), apart from the high SES private school where most boys (14/16; 88%) were boarders. The median age was 16 years (IQR 15–17). Overall, 82% students reported that they were Christian and 13% Muslim. Half the students (51%) were from the Baganda ethnic group; 6% were non-Ugandan. The majority of parents had completed secondary education (64% of mothers and 79% of fathers) (Table 1).

**Table 1:** Characteristics of participants by circumcision status^1^.

Characteristic	Total		Circumcised		Prevalence ratio	P-value
	N = 208	%	N = 123	% circ	95% CI	
SOCIODEMOGRAPHIC						
**Age group**						0.90
13–15 yrs	71	34.1	43	60.6	1	
16 yrs	52	25.0	30	57.7	0.95 (0.71–1.28)	
17 yrs	40	19.2	22	55.0	0.91 (0.65–1.27)	
18+ yrs	45	21.6	28	62.2	1.03 (0.76–1.38)	
**Religion**						<0.001
Catholic	64	30.8	36	56.3	1	
Anglican	54	26.0	32	59.3	1.05 (0.77–1.44)	
Born again	53	25.5	24	45.3	0.81 (0.56–1.16)	
Muslim	27	13.0	26	96.3	1.71 (1.36–2.15)	
Other	10	4.8	5	50.0	0.89 (0.46–1.71)	
**Ethnicity**						0.98
Muganda	105	50.0	61	59.2	1	
Non-Muganda	89	42.4	52	58.4	0.99 (0.78–1.25)	
Non-Ugandan	13	6.2	8	61.5	1.04 (0.66–1.64)	
**SES urban indicator**						0.23
Low	78	37.5	42	53.9	1	
High	130	62.5	81	62.3	1.16 (0.91–1.48)	
**Mother's educational level**						0.02
Did not complete secondary	73	36.1	35	48.0	1	
Completed secondary	129	63.9	84	65.1	1.36 (1.04–1.78)	
**Father's educational level**						0.03
Did not complete secondary	42	21.4	19	45.2	1	
Completed secondary	154	78.6	98	63.6	1.41 (0.99–2.00)	
**School type**						0.006
Government USE	112	53.9	70	62.5	1	
Government non_USE	53	25.5	29	54.7	0.88 (0.66–1.16)	
Private high SES	16	7.7	14	87.5	1.40 (1.11–1.77)	
Private low SES	27	13.0	10	37.0	0.59 (0.36–0.99)	
**Boarder or day student**						0.85
Day	175	84.1	103	58.9	1	
Boarder	33	15.9	20	60.6	1.03 (0.76–1.39)	
**Class**						0.21
Form 2	94	45.2	60	63.8	1	
Form 3	114	54.8	63	55.3	0.87 (0.69–1.08)	
**Father circumcised**						<0.001^§^
No	39	20.7	15	38.5	1	
Yes	84	44.7	67	79.8	2.07 (1.37–3.13)	
Don't know	65	34.6				
**Lifetime number of sexual partners**						0.19
None	119	60.1	69	57.9	1	
1	29	14.7	21	72.4	1.25 (0.95–1.64)	
2+	50	25.3	26	52.0	0.90 (0.66–1.22)	
**Age at first sex**						0.72
Not had sex	119	59.5	69	57.9	1	
Age <15 years	52	26.0	33	63.5	1.09 (0.85–1.41)	
Age > = 15 years	29	14.5	16	55.2	0.95 (0.66–1.37)	
**Ever had an HIV test**						0.005
No	96	47.3	48	50.0	1	
Yes	107	52.7	74	69.2	1.38 (1.09–1.75)	
**Do you drink alcohol?**						0.13
No	163	79.5	92	56.4	1	
Yes	42	20.5	29	69.1	1.22 (0.96–1.56)	
**Have you ever used an illegal drug?**						0.25
No	167	84.3	95	56.9	1	
Yes	31	15.7	21	67.7	1.19 (0.90–1.57)	

1 Excluding two boys with unknown circumcision status

There was strong evidence of differences in age and ethnicity by school (Appendix Table 1). The boys at the high SES schools were younger than those at low SES schools (median age 16 compared to 16.5 and 17 respectively; p = 0.01) and those at high SES schools had a lower proportion of ethnic Baganda students (34% and 31% compared to 55% and 72% respectively; p<0.001). Overall, 83 (41%) of boys reported having had sexual intercourse and 52 (26%) of all boys reported sexual debut before age 15. About half (n = 109; 53%) reported having had an HIV test (67% of those who reported having had sex). Of the 83 sexually active boys, 51 (61%) reported having had two or more sexual partners. The majority of boys reported that they did not drink alcohol (n = 165; 80%), and that they had never used an illegal drug (n = 169; 85%).

More than half the boys reported being circumcised (n = 123; 59%). Prevalence of circumcision ranged from 35% in the private low SES school to 88% in the private high SES school. In the univariable analysis (Table 1), being circumcised was strongly associated with Muslim religion and having a father who was circumcised (p<0.001) and was also associated with higher level of parent’s education, being in the private high SES school, and having ever had an HIV test.

Table 2 shows factors associated with circumcision (p<0.15) after adjusting for Muslim religion and school. Having had an HIV test is not included here as it is likely a result of attending for circumcision. In multivariable analysis, circumcision was independently associated with being at the private high SES school (aPR = 1.29, 95%CI 1.01–1.66 compared with the Government USE school), Muslim religion (aPR = 1.73, 95%CI 1.50–2.00), father having completed secondary education (aPR = 1.47, 95%CI 1.07–2.03), father being circumcised (aPR = 1.67, 95%CI 1.19–2.35) or not knowing father’s circumcision status (aPR = 1.44, 95%CI 1.00–2.06), and alcohol use (aPR = 1.29, 95%CI 1.05–1.59) (Table 2).

**Table 2:** Factors associated with circumcision status.

Characteristic	Adjusted PR^1^	95%CI	Adjusted PR^2^	95%CI
**Name of school**		P = 0.006		P = 0.002
Government USE	1		1	
Government non-USE	0.94	0.73–1.21	0.98	0.74–1.28
Private high SES	1.41	1.12–1.78	1.29	1.01–1.66
Private low SES	0.50	0.29–0.84	0.51	0.32–0.81
**Class**		P = 0.11		
Form 2	1	-		
Form 3	0.84	0.68–1.04		
**Ethnicity**		P = 0.53		
Muganda	1			
Non-Muganda	1.07	0.86–1.33		
**Religion**		P<0.001		P = 0.002
Non-Muslim	1		1	
Muslim	1.82	1.58–2.11	1.73	1.50–2.00
**Urban SES indicator**		P = 0.30		
Low	1			
High	1.13	0.89–1.42		
**Maternal education**		P = 0.02		
Did not complete secondary	1			
Completed secondary	1.32	1.04–1.69		
**Fathers education**		P = 0.01		P = 0.01
Did not complete secondary	1		1	
Completed secondary	1.46	1.06–2.00	1.47	1.07–2.03
**Father circumcised**		P<0.001		P<0.001
No	1		1	
Yes	1.70	1.23–2.34	1.67	1.19–2.35
Don’t know	1.30	0.90–1.86	1.44	1.00–2.06
**Ever had sex**		P = 0.54		
No	1			
Yes	1.07	0.86–1.33		
**Drinks alcohol**		P = 0.03		P = 0.04
No	1		1	
Yes	1.29	1.04–1.59	1.29	1.05–1.59

^1^ Adjusted for religion and school.

^2^ Adjusted for religion, school, father’s education, father’s circumcision status and alcohol use.

### Circumcision prevalence, knowledge, and perceptions

The proportion of boys with good knowledge of VMMC (defined as answering at least 4 of the 5 questions on knowledge correctly) was similar by circumcision status (68.3% versus 63.5%, PR = 1.08, 95%CI 0.88–1.31). Almost all boys knew that circumcision reduced the risk of HIV (95% of circumcised boys and 90% of uncircumcised boys) and 90% knew that circumcised men should still use condoms to prevent infection (Table 3). Knowledge about circumcision and risk of HIV in female partners, and risk of cervical cancer was weaker (with about 60% answering correctly, with no evidence of a difference in knowledge between circumcised and uncircumcised boys). The majority of boys had favourable perceptions about circumcision with 82% (including 81% of uncircumcised boys) reporting that it was “a good idea to get circumcised” (Table 3). There was evidence of high levels of acceptability among uncircumcised boys. For example, of the 85 uncircumcised boys, 67% stated they planned to get circumcised and 68% said they would get circumcised even if none of their friends did.

**Table 3:** Circumcision knowledge and perceptions among secondary school boys in Entebbe sub-district.

**CIRCUMCISION KNOWLEDGE**	**Total**	**n (%) with correct answer**	**PR**	**95%CI**
**Male circumcision reduces a man’s risk of getting HIV**				
Not circumcised	84	76 (90.5%)	1	-
Circumcised	123	117 (95.1%)	1.05	0.97–1.14
**Circumcised men don't need to use condoms**				
Not circumcised	84	76 (90.5%)	1	-
Circumcised	122	110 (90.2%)	1.00	0.91–1.09
**Male circumcision reduces the risk of HIV infection for female partners**				
Not circumcised	84	50 (59.5%)	1	-
Circumcised	121	73 (60.3%)	1.01	0.81–1.27
**Male circumcision reduces the risk of cervical cancer for female partners**				
Not circumcised	84	51 (60.7%)	1	-
Circumcised	121	67 (55.4%)	0.91	0.72–1.15
**I know where to go to get circumcised**				
Not circumcised	82	70 (85.4%)	1	
Circumcised	119	113 (95.0%)	1.11	1.01–1.23
**At least four questions correct**				
Not circumcised	85	84 (68.3%)	1	
Circumcised	123	54 (63.5%)	1.08	0.88–1.31
**FAVOURABLE CIRCUMCISION PERCEPTIONS**	**Total**	**% agree with answer**	**PR**	**95%CI**
**It is a good idea to get circumcised**				
Not circumcised	82	66 (80.5%)	1	-
Circumcised	123	103 (83.7%)	1.04	0.91–1.19
**Women prefer a circumcised man**				
Not circumcised	85	55 (64.7%)	1	-
Circumcised	123	87 (70.7%)	1.09	0.90–1.33
**I would get circumcised even if none of my friends did**				
Not circumcised	85	58 (68.2%)	1	-
Circumcised	120	100 (83.3%)	1.22	1.03–1.44
**I am comfortable talking about circumcision with friends**				
Not circumcised	84	64 (76.2%)	1	-
Circumcised	122	98 (80.3%)	1.05	0.91–1.22
**Circumcision increases a man's sexual pleasure**				
Not circumcised	85	36 (42.4%)	1	-
Circumcised	122	56 (45.9%)	1.08	0.79–1.48
**I have talked about circumcision with someone who has been circumcised**				
Not circumcised	85	70 (82.4%)	1	-
Circumcised	122	103 (84.4%)	1.03	0.91–1.16
**I have asked a question about circumcision in the past 2 months**				
Not circumcised	85	61 (71.8%)	1	-
circumcised	123	81 (65.9%)	0.92	0.76–1.10
**UNFAVOURABLE CIRCUMCISION PERCEPTIONS**	**Total**	**% agree with answer**	**PR**	**95%CI**
**Getting circumcised is dangerous**				
Not circumcised	84	15 (17.9%)	1	-
Circumcised	122	22 (18.0%)	1.01	0.56–1.83
**Circumcision is very painful**				
Not circumcised	85	52 (1.2%)	1	-
Circumcised	122	73 (59.8%)	0.98	0.78–1.22
**Circumcision healing process is very painful**				
Not circumcised	85	50 (58.8%)	1	-
Circumcised	122	68 (55.7%)	0.95	0.75–1.20
**Real men do not get circumcised**				
Not circumcised	85	11 (12.9%)	1	-
Circumcised	122	14 (11.5%)	0.89	0.42–1.86
**Circumcision reduces a man's sexual pleasure**				
Not circumcised	85	21 (24.7%)	1	-
Circumcised	123	27 (22.0%)	0.89	0.54–1.46
**I feel uncomfortable talking about circumcision**				
Not circumcised	85	21 (24.7%)	1	-
Circumcised	123	17 (3.8%)	0.56	0.31–1.00
**Circumcision reduces a man's sex drive**				
Not circumcised	85	16 (18.8%)	1	-
Circumcised	123	19 (15.4%)	0.82	0.45–1.50

### Intervention design

To adapt the Make The Cut intervention for Ugandan youth, the research team reviewed the curriculum, participated in the intervention procedures themselves, and provided insight on how the session could be improved and made specific to adolescent boys in Uganda. This resulted in revisions to include local Ugandan terminology and local VMMC service provider information and the inclusion of information regarding tetanus booster immunisation prior to VMMC, which is now required of all VMMC participants in Uganda. After revision, two recently circumcised peer educators were trained as Make The Cut coaches in a three-day workshop. The coaches learned and practiced delivering the intervention activities, including developing their “Coach’s Stories,” in which they shared their own personal experiences with VMMC. During the workshop, coaches field-tested Make The Cut at two local schools to gain practical experience. Observations by the research team showed coaches remained faithful to the plan for the session, conveyed accurate VMMC information, and were actively engaged with the youth participants.

**Fig 1:**
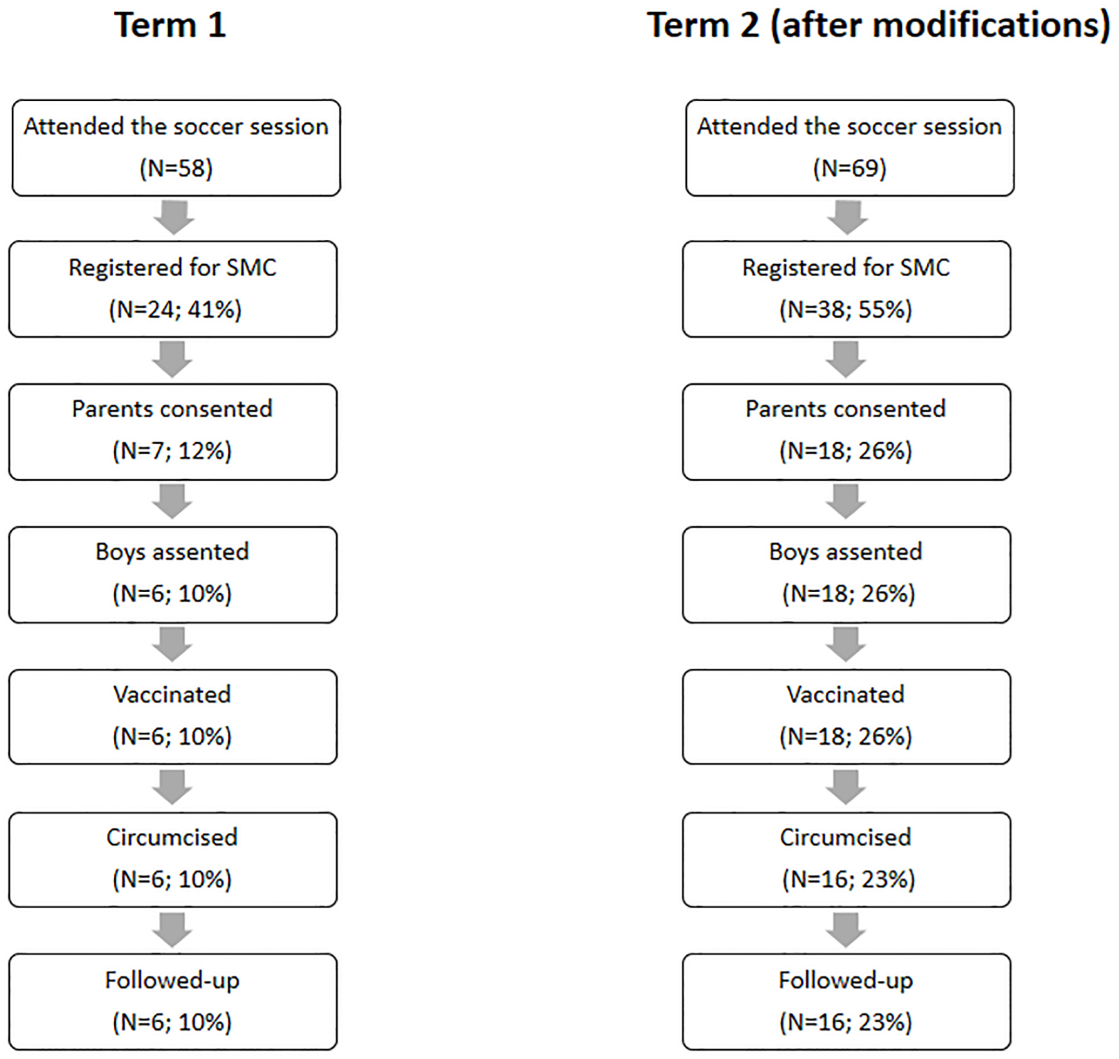
Progression of boys through the Make The Cut intervention program.

Coaches first implemented Make The Cut with 58 boys in the government low SES school, of whom 24 boys (41%) registered an interest in VMMC and only 6 (10%) were confirmed to have undergone circumcision (Fig 1). Challenges that contributed to the low confirmed uptake of VMMC included i) Coaches found it difficult to obtain parental consent for VMMC because of misconceptions about circumcision or about the time this would take from education; ii) School administrators only allowed the Make The Cut intervention to happen after normal school hours, resulting in low attendance; iii) Participants were most likely to undergo VMMC during the holiday time so they would not need to miss classes or exams but the intervention had initially been implemented in this school more than two months before the end of the term, resulting in some boys losing interest. Also, it was difficult for the coaches to communicate with boys during the holiday period to follow and encourage them.

Based on these findings, the following changes were made before implementation in the second school:

1. Coaches conducted home visits with boys who expressed an interest in being circumcised to actively engage with their parents. To assist in facilitating discussions with parents, coaches created and shared a short video of Make The Cut to both educate parents on VMMC and act as a discussion starter.
2. Coaches conducted in-person visits with school personnel to arrange for the Make The Cut sessions to happen during morning class periods.
3. Coaches implemented Make The Cut after exams, but before the end of the term. This reduced the time between implementation and uptake and avoided the need for communication with the boys during holiday time.

Coaches implemented these changes in the second school (the low SES private school), where we saw an encouraging increase in VMMC referral and uptake compared to the first school (p = 0.06), with 38 of the 69 boys who attended the session (55%) registered for VMMC and 16 (23%) confirmed to have undergone the procedure (Fig 1).

### Qualitative results

The qualitative findings are based on data from boys who attended the Make The Cut intervention. Three key themes emerged: 1) role of social support, 2) reasons for and against circumcision and 3) future VMMC considerations.

#### Role of family and social support

The attitudes of family and peers were central to the decision to become circumcised in this adolescent population. The circumcised participants in the Make The Cut intervention indicated having benefitted from various sources of social support from family and peers in the form of information and encouragement. Even before participants met the coaches, they cited particularly parents and other relatives as trusted sources from whom they drew support,

*“I first heard from home when they wanted to take us for circumcision forcefully against our will. So I left home and when the whole issue was over I went back home and they did not bother me about that again. So when I went to my father’s place he explained to me about it more and he said that if you are not circumcised you can easily get HIV but if you are circumcised then you reduce chances of getting the virus”*.

(IDI Participant 05, Circumcised).

#### Role of the coaches

While family and peers played an important role in preparing the participants for circumcision, the decision to circumcise was prompted by coaches. Participants acknowledged this crucial role of coaches, which entailed the explanation of information regarding circumcision including the healing process and the discussion of the myths or misconceptions that had held participants back from deciding to circumcise. Participants were satisfied with the support from coaches not only before but also after circumcision, as the coaches followed up with the participants in the aftermath of circumcision.

“[The coaches] were teaching us some things and even they could call and ask me whether I am okay and they even used to give me transport to go to the hospital. The whole process was okay because I felt comfortable with it and whatever you could ask they could reply you.”

(IDI Participant 02, Circumcised).

#### Reasons for and against circumcision

The main reasons for becoming circumcised were the health benefits of improving genital hygiene and reducing the risk of being infected with HIV [15, 16], and a main barrier was fear of pain [15–17]. In addition, those who participated in the Make The Cut intervention but declined circumcision attributed this to loss of contact with their coaches.

“We were given phone contacts by the coaches but then I lost mine so I couldn’t contact anyone. So when I tried looking for the coach’s contacts I realized I had lost them and so I gave up and later afterwards my friend came and told me that he had finished getting circumcised.”

(IDI Participant 03, Uncircumcised).

The influence of the social and family circles was also clear, including some family members who threatened not to care for the boys if they underwent the procedure.

*“It [not circumcising] was because of my sister. My mum used to go for work and would come back late at around midnight and my sisters were in senior five and they told me that if I go for circumcision they will not look after me. They said that I will be on my own and stay home alone and no one will either give the medicine, nor cleaning you”*.

(IDI Participant 04, Uncircumcised).

#### Influencing future VMMC decision-making?

All the boys who remained uncircumcised indicated a willingness to circumcise in future when they have overcome the challenges that had rendered it difficult to circumcise. Some of them were willing to circumcise after their exams while others needed further assurance that the procedure would not be painful.

*“After I finish my last examination paper I will go for circumcision. I want to heal very fast because I have other things I would like to do thereafter. Like in one month I will have healed so that I do other things”*.

(IDI Participant 02, Uncircumcised).

## Discussion

The study shows that secondary schools can provide a suitable venue for VMMC interventions, and co-ordination of VMMC provision with school-based interventions such as Make The Cut could improve uptake. The relatively high VMMC prevalence among adolescent boys in this study is encouraging evidence that Uganda is making progress towards the UNAIDS target of 80% VMMC coverage. However, to reach the target of 80% coverage, there is a need for demand-creation interventions that provide motivation specifically to males who have not yet chosen to undergo circumcision despite widespread media campaigns, demand-creation initiatives, and social pressure. The VMMC uptake seen in our study following the modified intervention suggests that Make The Cut can be effective in reaching uncircumcised boys where baseline prevalence is already high. These findings are consistent with the study on Make The Cut among Zimbabwean adolescents, in which 48% of participants self-reported as circumcised at baseline, and 12% underwent the procedure following Make The Cut [9]. Additionally, Make The Cut aligns with PEPFAR VMMC age guidelines, which prioritize VMMC programmes targeted at males aged 15–29 years [18].

The importance of including the family and community was a key finding from our qualitative data, and reflects similar findings from a school-based VMMC promotion program in South Africa, which also reported the critical role of the community and family involvement for VMMC uptake among school-based youth [10]. Our study also showed that the inclusion of in-person follow-up with students and families in the second phase of implementation of Make The Cut contributed to the increased uptake of VMMC. The continued interaction strengthened the relationship between the coach and student and appeared to influence the participants’ decision to undergo the procedure. Similarly, a recent study in Uganda and South Africa found in-person follow-up with a lay counsellor contributed to significant VMMC uptake [19]. However, in-person visits are potentially costly and can create logistical and administrative challenges, which could make this component of the intervention difficult to replicate at scale. In addition to the sport-based interpersonal model utilized in Make The Cut, impact evaluations on male circumcision uptake have found intervention components such as small incentives [20, 21] and SMS follow-up [22] can be effective in increasing demand for VMMC. Additional research is needed to determine whether SMS communication between coach and participant can enable the continued relationship in a way that is more cost-effective than in-person follow-up.

In this study, self-reported prevalence of VMMC among adolescent males was almost double the adult VMMC prevalence in Ugandan (59% vs 33%) [2]. This finding supports a recent study in Rakai District, Uganda, which found that almost half of boys aged 15–17 years were circumcised (49.3% among all boys; 40.2% among non-Muslim boys) (Gray et al, personal communication). These findings reflect general trends in several other UNAIDS/WHO circumcision priority countries, which show adolescent males are generally more willing to undergo circumcision than adult males [23].

This study had a number of limitations. As this was a feasibility study, the study was small, and there was no control group which means that we cannot attribute the observed VMMC uptake directly to the Make The Cut intervention. Similarly, although the uptake of VMMC increased after our curriculum modification, we cannot attribute this increased uptake to the modification directly. A further limitation is that VMMC uptake was only measured for three months following the intervention, and it is possible that the intervention resulted in boys deciding to become circumcised after completion of our study.

This study showed that the modified Make The Cut intervention may be effective to increase uptake of safe male circumcision in this population. However, the intervention is time-intensive, and further work is needed to assess the cost-effectiveness of the intervention conducted at scale.

## Supporting information

S1 File In-depth interview guides for boys who became circumcised during the Make The Cut intervention. (PDF)

S2 File In-depth interview guides for boys who did not become circumcised during the Make The Cut intervention. (PDF)
