# Supplementary material for: Soccer-based promotion of voluntary medical male circumcision: A mixed-methods feasibility study with secondary students in Uganda
Source: PLoS One. 2017 Oct 9;12(10):e0185929. doi: 10.1371/journal.pone.0185929 (PMC5633183; doi:10.1371/journal.pone.0185929)
Supplement: S1 File — (PDF) [file pone.0185929.s001.pdf]

Safe Male Circumcision (SMC), Male Students (14-17 years)

In Depth Interview Guide, For Use 6 Weeks-Post SMC

Opening Questions

Opening questions are intended to build rapport and gradually lead into the key questions

Questions	Probes/follow-ups
1. What is your favourite soccer team?<br>2. Who is your favourite player? Why?	In Uganda? In England or Europe?

Key Questions – 1. Opening demographics

Questions	Probes/follow-ups
1. Which class and stream are you in?	How old are you?

Key Questions – 2. SMC information sources, decision-making, social context

Questions	Probes/follow-ups
1. We recently talked to you about safe male circumcision (SMC)? Was this the first time you heard about SMC? If not, where did you hear about it.	Clinic outreach activities?<br>Public education campaigns?<br>Public community meetings?<br>Discussion among friends?

2. What are the main reasons you decided to get circumcised?<br><br>3. Whom did you talk to about circumcision?<br><br>4. How helpful was the coach in your decision to become circumcised?<br><br>5. Who else helped you with your decision?	
6. What was your first reaction when you heard about circumcision?<br><br>7. What kinds of concerns or fears did you have?<br><br>8. How has your opinion about circumcision changed since being circumcised?	

Key Questions – 3. Experiences of SMC process – accompaniment and counselling	
**Questions**	**Probes/follow-ups**
1. Now, let's talk about what happened on the day you went for circumcision. Who accompanied you to the clinic on the day of the procedure?	How influential was the person who accompanied you in making the decision to get circumcised?<br><br>How were you feeling when you arrived at the clinic?
2. Do you remember attending a counselling session before getting circumcised? What information did you receive about circumcision during the counselling session?<br><br>3. Was this information helpful? Why or why not?<br><br>4. During counselling, did you learn any new information about SMC? Did it change the way you felt about circumcision? How?<br><br>5. After the counselling, did you have any further questions about the SMC procedure? What additional information did you want?	Were the benefits of SMC explained to you? What were the most important benefits to you? Why?<br><br>Were the risks of SMC explained to you? Which of those worried you the most? Why?<br><br>Did the counsellor discuss sex and HIV prevention with you? If so, what did the counsellor say?

Key Questions – 4. Experiences of SMC process – procedure

Questions	Probes/follow-ups
1. Looking back on your experience of the circumcision procedure, how did it compare to what you expected?<br><br>2. How would you explain to a friend what to expect from the procedure?	Was it more or less painful?<br><br>What was similar to what you expected?<br><br>What was different to what you expected?<br><br>What was the easiest part about the procedure?<br><br>What was the most difficult part about the procedure?

Key Questions – 5. Experiences of SMC process – healing

Questions	Probes/follow-ups
1. Looking back on your experience of healing after the circumcision procedure, how does it compare to what you expected?<br><br>2. How would you explain to a friend what to expect from the healing process?	Was it more or less painful?<br><br>What was similar to what you expected?<br><br>What was different to what you expected?<br><br>What instructions did you receive to take care of the wound? Were these helpful?
3. Have you recommended circumcision to a friend?<br><br>If not, why not?	How was it received?

Thank you for your participation. Your involvement is really important to us, and we appreciate you sharing your personal experiences.

Before we end, do you have any questions for me about safe male circumcision, the interview, or other information we have discussed?
