# Supplementary material for: Soccer-based promotion of voluntary medical male circumcision: A mixed-methods feasibility study with secondary students in Uganda
Source: PLoS One. 2017 Oct 9;12(10):e0185929. doi: 10.1371/journal.pone.0185929 (PMC5633183; doi:10.1371/journal.pone.0185929)
Supplement: S2 File — (PDF) [file pone.0185929.s002.pdf]

Safe Male Circumcision (SMC), Male Students (14-17 years)

In Depth Interview Guide, For Use 6 Weeks-Post SMC

Opening Questions

Opening questions are intended to build rapport and gradually lead into the key questions

Questions	Probes/follow-ups
1. What is your favourite soccer team?<br>2. Who is your favourite player? Why?	In Uganda? In England or Europe?

Key Questions – 1. Opening demographics

Questions	Probes/follow-ups
1. Which class and stream are you in?	How old are you?

Key Questions – 2. SMC information sources, decision-making, social context

Questions	Probes/follow-ups
1. How did you first hear about safe male circumcision (SMC)? When did you first hear about SMC?	Clinic outreach activities?<br>Public education campaigns?<br>Public community meetings?<br>Discussion among friends?

2. Why did you choose not to get circumcised?<br><br>3. Whom did you talk to about circumcision?<br><br>4. Whose views were most influential in making the decision not to get circumcised?	Improved sexual performance? Decreased HIV risk? Hygiene?<br><br>What was the most important factor in your decision to get circumcised?<br><br>What did you hear about circumcision from your parent/guardian? Friends? Other people (Uncle, teacher, priest, community leader, etc.)?
5. What was your first reaction when you heard about circumcision?	What kinds of concerns or fears did you have?
6. Are you considering getting circumcised?	
7. What would it take for you to decide to get circumcised?	

Thank you for your participation. Your involvement is really important to us, and we appreciate you sharing your personal experiences.

Before we end, do you have any questions for me about safe male circumcision, the interview, or other information we have discussed?
